# The immediate and short-term impact of COVID 19 infections on nurses in a UAE hospital

**DOI:** 10.1186/s12912-022-00979-y

**Published:** 2022-07-19

**Authors:** Khaled Al Qawasmeh, Nabeel Al Amiri, Salma Omar Al Nuaimi

**Affiliations:** Department of Nursing, Tawam Hospital, Post Box 15258, Al Ain, UAE

**Keywords:** COVID 19, COVID 19 stigma, Nurses, Physical, Psychological, Social, Self-esteem, UAE

## Abstract

**Background/Aims:**

Nurses were on the front line against the COVID 19 pandemic, fighting to save human lives. Many nurses sacrificed their well-being and social life to win the battle. In consequence, many nurses have been infected with the virus around the Globe. This study aims to determine the immediate and short-term physical, psychological, and social impact of COVID 19 infection on nurses and midwives retrospectively. On the other hand, it aims to find the effect of COVID 19 stigma on the self-esteem of the infected nurses.

**Methods:**

To achieve this goal, the authors used an exploratory, mixed-method design with a sample of nurses and midwives working in a tertiary hospital in UAE who has been infected with the COVID 19 virus and recovered. The authors have used the qualitative results to explain and interpret the findings of the quantitative findings. The data were collected through distributing the quantitative survey to participants and then it was followed by conducting semi-structured interviews.

**Results:**

The careful exploration of the experiences of nurses infected with the COVID 19 virus suggested a simple model that manages the patients in hospitals and at homes, including improving self-efficacy and patient coping, providing the basic nursing skills to patients and families, providing continuous psychological support, and providing high standards of health care.

**Conclusion:**

The new suggested model will maintain a positive status of wellbeing amongst infected patients during the infection time and in 3–6 months after the infection.

## Background

The Severe acute respiratory syndrome coronavirus or COVID-19 was first discovered in Wuhan, China, in Jan 2020. Soon after, many countries around the world reported positive cases, including the UAE. The pandemic crisis has changed the working environment and job requirements dramatically.

One study conducted in Helsinki University Hospital by [21] revealed that 4.7% of the participants who were tested positive for COVID 19 were healthcare workers, which makes the percentage of infection higher than that of the general population (0.3%). More than half (53.6%) of infections were confirmed or likely occupational either from colleagues or while treating suspected or confirmed COVID-19 patients. Another rabid review identified the risk factors for COVID 19 infection among healthcare workers as the lack of personal protective equipment, exposure to infected patients, work overload, poor infection control, and pre-existing medical conditions [18].

Based on official figures and media reports from a limited number of countries, the International Council of Nurses [29] announced that more than 230,000 healthcare workers contracted the COVID-19 virus, and more than 600 nurses died from the disease worldwide in the first 5 months. To cover all the world’s countries, the ICN extrapolated the previous numbers and revealed that around 450,000 of the world’s over six million cases could be among healthcare workers. Therefore, Dunham [12] considered nursing as one of the most dangerous jobs in the world.

In the context of Tawam Hospital and UAE, nurses responded to the government call to stand against the new pandemic; they worked with positive cases inside hospital wards, such as medical and intensive care units, and in quarantine centres, such as hotels. Also, they worked in public areas, including borders, airports, and public areas.

Many nurses reported symptoms and confirmed later as positive COVID 19 cases. The nurse's experiences during the infection time were hard and challenging associated with fear, stress, and uncertainty. We have no idea about the immediate and short-term impact of COVID 19 infections on nurses. Therefore, this study aims to determine the immediate and short-term physical, psychological, and social impact of COVID 19 infection on nurses and midwives retrospectively and determine the effect of the COVID-19 infection stigma on the self-esteem of the infected nurses at a selected tertiary hospital in the UAE.

### Literature review

Coronavirus is one of seven types of known human coronaviruses, like the MERS and SARS coronaviruses. This large family of viruses causes illness in humans and animals and no specific treatment is approved for COVID19 infection to date [20].

The WHO [31] reported that people with COVID 19 develop signs and symptoms, including mild respiratory symptoms and fever, on an average of 5–6 days and a range of 1–14 days. The report summarized the physical symptoms of COVID-19. The report highlighted that the typical signs and symptoms include fever (87.9%), dry cough (67.7%), fatigue (38.1%), sputum production (33.4%), shortness of breath (18.6%), sore throat (13.9%), headache (13.6%), myalgia or arthralgia (14.8%), chills (11.4%), nausea or vomiting (5.0%), nasal congestion (4.8%), diarrhea (3.7%), and hemoptysis (0.9%), and conjunctival congestion (0.8%). According to Jamil, et al., [16] other symptoms and complications, including the loss of taste or smell, stroke, and skin rash are also included in the COVID 19 symptoms list.

According to the WHO [31] report, the symptoms are non-specific and they can range from no symptoms in rare cases, mild and moderate symptoms, which include non-pneumonia and pneumonia cases (80%), severe disease, which includes dyspnea, the respiratory rate more than 30 per minute, blood oxygen saturation less than 93%, and lung infiltrates of more than 50% of the lung field in 24 to 48 h (13.8%), and critical symptoms, which include respiratory failure, septic shock, and multiple organ dysfunction (6.1%). Furthermore, the WHO [31] highlighted that people aged over 60 years and those with underlying conditions such as hypertension, diabetes, cardiovascular disease, chronic respiratory disease, and cancers are at the highest risk for severe disease and death.

WHO (EPI-WIN) [32] mentioned that people usually recover from COVID 19 in 2 to 6 weeks. For some people, some symptoms may linger or recur for weeks or months following initial recovery in people with mild disease. Those symptoms include fatigue, cough, congestion or shortness of breath, loss of taste or smell, headache, body aches, diarrhea, nausea, chest or abdominal pain, and confusion. However, people are not infectious during this time.

The (ICN) [30] reported that the total number of the reported COVID 19 deaths was increased to 2,262 in nurses in 59 countries, and COVID 19 infections were reached more than 1.6 million among healthcare workers in 34 countries as of 31 December 2020. The ICN estimated that around 10% of all confirmed COVID-19 infections are among healthcare workers. Al Maskari [1] found in a cross-sectional study in health care workers in Oman that more than three-quarters of the infected health care workers had no chronic diseases or risk factors for severe COVID-19, while 7% had hypertension, 11% had diabetes mellitus, and 3% had other chronic diseases. The study also found that the most common acquisition of COVID-19 among health care workers was from the community (61.3%), by hospital acquisition (25.5%), and no clear source was identified for the rest (13.2%) of cases. Among those who acquired COVID-19 in the hospital, around one quarter (35%) acquired the infection from a confirmed positive colleague and around two quarters (65%) from exposure to infected patients. An internal unpublished report from Tawam hospital (the study location) presented that a total of 113 nurses were infected with COVID 19 viruses and the number of deaths was zero [28].

Gheysarzadeh, et al., [14] highlighted that despite nurses having enough skill and knowledge, they can be infected quickly as the result of their exposures to infected patients. However, the study showed that receiving the necessary care and treatment at home was a good experience for nurses and can be used for some cases.

On the other hand, Literature highlighted several impacts of the COVID 19 pandemic on people, including psychological, social, well-being, self-esteem, and others. For example, a report by Simetrica-Jacobs [27] from the UK about the wellbeing costs of COVID19 in April 2020 compared to March and April 2019 concluded that the health, social and economic impacts of COVID-19 and social distancing are associated with large reductions in a range of wellbeing in terms of life satisfaction, happiness, sense of worthwhile, and anxiety and increases in psychological distress, with some evidence that the impacts are more severe for women and ethnic minority groups. Dagnino, et al., [10] found several psychological impacts of the quarantine, including various concerns (67%) and anxiety (60%), and concerns about the future, including concerns about general health (55.3%), employment (53.1%), and finances (49.8%). More, Ripon, et al., [25] claimed that the prevalence of depression and post-traumatic stress disorder (PTSD) is 85.4% among those who had home quarantine and 94% among those who had institutional quarantine in Bangladesh.

Nevertheless, Otu, et al., [22] pointed out in a literature review that the mental health care of patients, health professionals, and communities is likely under-addressed during COVID 19 pandemic, which could raise the major medium and long-term consequences and, accordingly, a proactive longer-term strategy rather than short-term crisis responses is desirable. Also, Dagnino, et al., [10] found that almost half of the participants (43.8%) felt they would need emotional support after this pandemic.

In sequence, Literature highlighted a new phenomenon COVID 19 stigma and discrimination. The Merriam-Webster dictionary [17], defines stigma as a mark of shame or discredit. CDC [5] pointed out that stigma related to COVID 19 is associated with the lack of knowledge about how the virus spreads, a need to blame someone, fears about disease and death, and common rumors and myths about the disease. The CDC [5] highlighted several groups of people who may experience stigma during the COVID-19 pandemic, including 1) certain racial and ethnic minority groups, 2) people who infected and recovered, 3) emergency responders or healthcare providers, 4) other frontline workers, such as grocery store clerks and delivery drivers, 5) people having disabilities or developmental or behavioral disorders that make them unable to follow the protection instructions, 6) people who have underlying health conditions that cause a cough, and 7) people living in groups. Therefore, those groups of people could experience discrimination in form of rejection by other people, denying providing specific services to them, such as healthcare, education, housing, or employment, verbal abuse, and physical violence CDC [5].

Ramaci, et al., [23] found that stigma positively predicted burnout and fatigue and negatively predicted satisfaction among frontline care providers working with patients infected with the COVID-19 in a large hospital in Italy. In India, Yadav, et al., [33] reported that 70% of a sample of health care provider perceived some kind of stigma, 50% perceived some form of stigma in their residential colony, 46% observed change in behaviour of their neighbours, and round 20% experienced rude behaviour or harassment from neighbour/landlord. Additionally, Munson [19] claimed that the positive experience leads to high self-esteem, while the experience of failure or rejection leads to low self-esteem. However, Dimitriadou–Panteka, et al., [11] reported that self-esteem correlates perfectly with the way one experiences reality, no matter true or false perceptions.

In 2020 and 2021, academics published a large number of articles. Most of these articles were relevant to the impact of the COVID 19 pandemic on people and health care providers. Nevertheless, the number of articles that addressed the experience of people and health care providers, who were infected with the COVID 19 virus, was less.

### Study questions

This study aims to answer two main questions as follows:

1. What are the immediate and short-term physical, psychological, and social impacts of COVID 19 infection on the infected nurses and midwives at Tawam Hospital?
2. What is the effect of the COVID-19 stigma on the self-esteem of the infected nurses?

### Study objectives

1. To determine the immediate and short-term impacts of the COVID-19 infection on the physical, psychological, and social impact of the nurses and midwives.
2. To find the effect of COVID-19 stigma on the self-esteem of the infected nurses.

### Significance of the study

As COVID 19 crisis is still ongoing, a second stronger wave is currently beating the world. The number of cases was dramatically increased and exceeded 38,000,000 until the moment of writing this paper. Experts are becoming more certain that the crisis will last for longer periods and will cause more harm to people, including deaths.

Hence, understanding nurse's experience with the COVID 19 infection could help other nurses and health care providers in the field to gain the courage and the confidence to continue fighting the disease until we have a great victory. Also, the study could help nurses and other healthcare providers in other similar crises in the future.

This study will provide important information about the immediate and short-term physical, psychological, and social impact of COVID 19 infection on nurses and midwives and about the effect of COVID 19 stigma on the self-esteem of the infected nurses since it is the first study, at least in the MENA area, to explore this concept.

## Methodology

### Methods, study design

The authors used an exploratory, mixed methods research design. According to Creswell & Plano [9], the mixed methods research design focuses on collecting, analysing, and mixing both quantitative and qualitative data through using all the available tools to provide a better understanding of the research problem. Bryman [4] suggested that the combining of both quantitative and qualitative research allows the researcher to offset the weaknesses of each design and draw on the strengths of both. Also, Bryman [4] suggested using qualitative data to illustrate quantitative findings, often referred to as putting meat on the bones of dry quantitative findings to improve the usefulness of findings.

In this study, the authors have used the sequential explanatory approach to mixed methodology guided by Creswell [8] where the quantitative phase was conducted first and then followed by the qualitative phase. The authors have used the qualitative results to explain and interpret the findings of the quantitative findings.

The data were collected through distributing the quantitative survey to participants and then it was followed by conducting semi-structured interviews.

### The sample

The authors adopted the whole sampling techniques to collect the required data for the first quantitative phase of the study. The authors targeted all Tawam Hospital nurses who were infected and recovered from COVID-19 between February and July 2020.

For the second qualitative phase, the authors conducted semi-structured interviews with an extreme case sample of nurses who were affected negatively by the COVID 19 infection and included in the primary sample. According to Creswell [7], 5 – 25 cases are required for phenomenological (lived experiences) studies; therefore, the authors interviewed a sample of 10 nurses to cover the qualitative part of the study.

### Inclusion and exclusion criteria

The authors included nurses working at Tawam Hospital who were infected with COVID-19 and recovered. The authors excluded those who infected after June 2020.

### Data collection tool

The study included two surveys: the first is quantitative, and the second one is qualitative.

The first survey included several parts that collected information about the respondents, the physical symptoms, emotions, coping, and social status during COVID 19 infection period and 3–6 months after. The study used a Likert scale to rate the participant's responses for selected statements on scales that vary from 1 (did not experience at all) to 5 (the experience was extremely strong). Furthermore, the survey explored the impact of COVID-19 infection on the physical, psychological, and social status of infected nurses at two points of time; first during the infection period (immediate) and after 3–6 months (short-term).

The second survey included 3 open-ended questions that aimed to explore the effect of COVID 19 infection on the infected nurse's life through conducting semi-structured interviews with selected nurses of the primary sample. The open-ended questions included:

- Q1: Please, describe if COVID 19 infection has a significant effect on you?
- Q2: What did you learn from that experience?
- Q3: Describe if COVID 19 infection affected your future goals?

The two surveys were prepared primarily by the investigator to serve the objectives of the study based on the literature review. Also, the authors got some benefits from the ‘International Stress Management Association’ (ISMA) UK [15] stress questionnaire, the Gallup-Healthways [13] well-being index, and Rosenberg [26] self-esteem scale.

### Ethical Consideration

The research was approved by the Department of Health (DOH) COVID19 IRB Committee for research Abu Dhabi, UAE. The study was carried out per the International Conference for Harmonization (ICH), and Good Clinical Practice guidelines. Approval for Informed consents for participation in this study was granted by the Abu Dhabi Technical and Scientific Human Research Ethics Committee, a central research ethics committee at the department of health Abu Dhabi, UAE. The authors adhered to the research ethics all the time.

### Data analysis and statistical method

#### Analysis of the quantitative part

The quantitative part of the study was analysed using the Statistical Package for the Social Sciences V.23. Thematic analysis was used for the qualitative part.

## Results

For the first part of the study, the authors received responses from 22 nurses out of 113 infected nurses in November and December 2020. The analysis of the demographic data showed that the majority of the respondents were between the ages of 31 and 40 years, females, staff nurses, holding bachelor degrees, and working in in-patient's units. See Table 1 for details.

**Table 1:** Demographic information of the respondents

Variable	Category	Frequency	Percent	Cumulative Percent
Age	24—30	1	4.5	4.5
	31—40	14	63.6	68.2
	41—50	5	22.7	90.9
	51—60	2	9.1	100.0
Gender	Female	19	86.4	86.4
	Male	3	13.6	100.0
Education	Bachelor	19	86.4	86.4
	Diploma	2	9.1	95.5
	Master	1	4.5	100.0
Position	CN	1	4.5	4.5
	PN	2	9.1	13.6
	SN	19	86.4	100.0
Unit	ER	3	13.6	13.6
	Inpatient	13	59.1	72.7
	OR	2	9.1	81.8
	Outpatient	4	18.2	100.0
Total		22	100.0	

### Reliability analysis

To assess the reliability of the data collection tool, Cronbach's alpha was calculated and presented in Table 2. The Cronbach's alpha for the scales is above 6.0 (the acceptable value for the Cronbach's alpha) except for one subscale. However, the value of Cronbach's alpha for the entire tool, including all subscales, is 0.945.

**Table 2:** Reliability analysis

Variable	Scale	Cronbach’s Alpha		
Physical symptoms	Physical symptoms during COVID 19 infection	0.886	0.890	0.945
	Physical symptoms 3–6 months after COVID 19 infection	0.792		
Emotions	Emotions during COVID 19 infection	0.911	0.942	
	Emotions 3–6 months after COVID 19 infection	0.953		
Social status	Social status during COVID 19 infection	0.628	0.664	
	Social status 3–6 months after COVID 19 infection	0.454		
Daily activities	Daily activity during COVID 19 infection	0.729	0.866	
	Daily activity 3–6 months after COVID 19 infection	0.778		
Self-esteem	Self-esteem during COVID 19 infection	0.87	0.856	
	Self-esteem 3–6 months after COVID 19 infection	0.898		

### Descriptive statistics

**Fig. 1:**
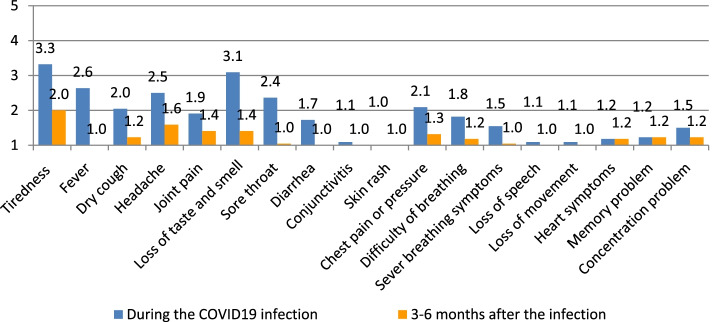
Description of the physical symptoms among participants during and 3–6 months after the COVID 19 infection

**Fig. 2:**
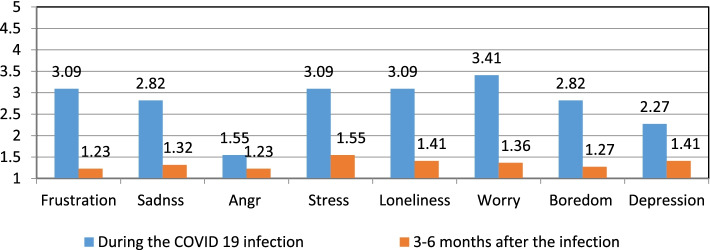
Description of the emotions among participants during and 3–6 months after the COVID 19 infection

**Fig. 3:**
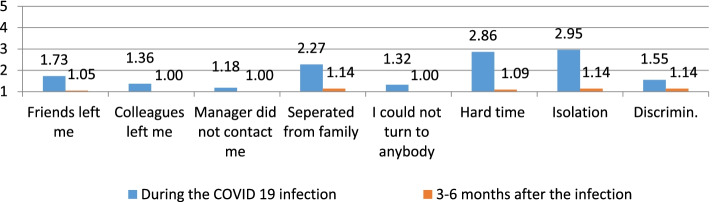
Description of the social status of participants during and 3–6 months after the COVID 19 infection

**Fig. 4:**
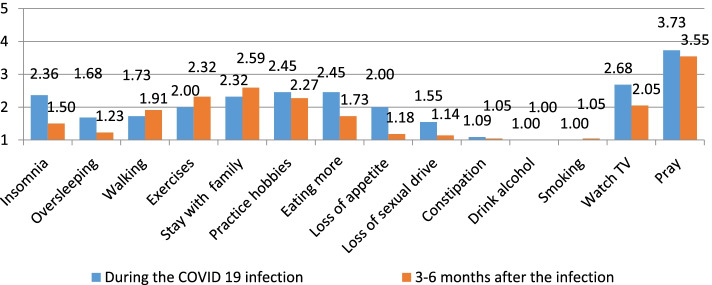
Description of the daily activities of participants during and 3–6 months after the COVID 19 infection

**Fig. 5:**
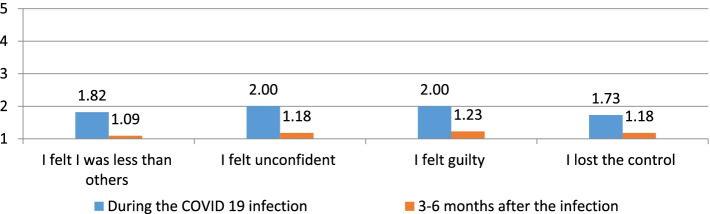
Description of the self-esteem of participants during and 3–6 months after the COVID 19 infection

To determine the immediate and short-term physical, psychological, and social impact of nurses and midwives due to the COVID-19 infection. The authors calculated the averages (means) of the responses, as suggested by Boone and Boone [3], of the individual items that make up one scale that is more reliable than the individual items and then presented those averages in a bar chart. This calculation is repeated for the rest of the scales (Figs. 1, 2, 3, 4, and 5). The data could be interpreted as follows: 1 indicates that all respondents rated the scale item 1 (strongly disagree), 1.01—1.8 indicates that the majority of respondents rated the scale item as very low, 1.81—2.60 indicates that the majority of respondents rated the scale item low, 2.61—3.40 indicates that the majority of respondents rated the scale item moderately, 3.41—4.20 indicates that the majority of respondents rated the scale item high, and 4.21—5.00 indicates that the majority of respondents rated the scale item very high Arcenas [2].

Regarding the physical symptoms, Fig. 1 shows that tiredness, fever, and loss of smell and taste were reported moderately, dry cough, headache, joint pain, sore throat, chest pain, and difficulty of breathing were low, and the other symptoms, including severe breathing problems, heart symptoms, and loss of movement were very low among the respondents during the infection time. See also the responses 3–6 months after the COVID 19 infection.

Regarding the respondent's emotions during the COVID 19 infection, the worry was high; frustration, sadness, stress, loneliness, and boredom were moderate; depression was low; anger was very low (Fig. 2). See also the responses 3–6 months after the COVID 19 infection.

About the social status, isolation and spending a hard time were reported moderately, while the other items were low to very low (Fig. 3). See also the responses 3–6 months after the COVID 19 infection.

Regarding daily activities, respondents reported watching TV and praying moderately, while the other activities were low to very low. Some activities such as smoking and drinking alcohol were not reported at all (Fig. 4). See also the responses 3–6 months after the COVID 19 infection.

The influence of COVID 19 on the respondent's self-esteem was low to very low, as presented in Fig. 5. See also the responses 3–6 months after the COVID 19 infection.

### Inferential statistics

This study ended with two data sets; one data set presents the means and standard deviations for all variables, including the physical symptoms, emotions, social status, daily activities, and self-esteem during the COVID 19 infection and the second set presents the same variables 3–6 months after the infection. Table 3 presents the means and standard deviations of all variables (scales) before and 3–6 months after the infection.

**Table 3:** The means and standard deviations for all variables (Scales)

Variable		Mean	N	SD	Std. Error Mean
Pair 1	Physical symptoms/During	1.85	22	.69	.15
	Physical symptoms/After	1.22	22	.29	.06
Pair 2	Emotion/During	2.77	22	1.13	.24
	Emotion/After	1.35	22	.77	.16
Pair 3	Social Status/During	1.90	22	.63	.13
	Social Status/After	1.07	22	.14	.03
Pair 4	Daily activity/During	2.00	22	.60	.13
	Daily activity/After	1.75	22	.55	.12
Pair 5	Self-esteem/During	1.89	22	1.12	.24
	Self-esteem/After	1.17	22	.54	.12

The normality test, specifically the Shapiro–Wilk test that is more appropriate when the sample size is less than 50 since our sample size is less than 30, is a prerequisite before going further. If the sample is normally distributed, then the paired t-test could be used for the comparative analysis. Otherwise, the paired samples Wilcoxon test could be an alternative. To test the normality, the two data sets, which present the total scores of all variables for each participant during and 3–6 months after the infection, are reduced to one new data set by subtracting the second data set from the first one. Then, the new data set is analyzed to find the results of the Shapiro–Wilk tests (Table 4).

**Table 4:** The tests of normality

	Kolmogorov-Smirnov^a^			Shapiro–Wilk		
	Statistic	df	Sig	Statistic	df	Sig
Difference	.169	22	.105	.934	22	.145

^a^ Lilliefors Significance Correction

In reference to the above table, the Sig. value of the Shapiro–Wilk test is 0.145 (> 0.05), which indicates that the data is very close to the normal distribution. Accordingly, the authors conducted the comparative analysis using the paired t-test, which usually measures the difference between two variables for the same sample separated by time.

The SPSS software also performs correlation analysis when comparing two sets of data for the same variables to find how strongly the two variables are associated with one another. Table 5 presents a moderate positive correlation between all pairs.

**Table 5:** The correlations of the paired samples

Paired Samples		N	Correlation	Sig
Pair 1	Physical symptoms (During—after)	22	.394^a^	.069
Pair 2	Emotion (During – after)	22	.607 ^a^	.003
Pair 3	Social Status (During—after)	22	.491 ^a^	.020
Pair 4	Daily activity (During – after)	22	.708 ^a^	.000
Pair 5	Self-esteem (During – after)	22	.466 ^a^	.029

^a^Correlation Coefficient: 0 (no correlation), 0 to 0.3 or 0 to -0.3 (weak positive or negative), 0.3 to 0.7 or -0.3 to -0.7 (moderate positive or negative), and 0.7 to 1.0 or − 0.7 to − 1.0 (strong positive or negative) (Ratner, 2009 [24])

Furthermore, Table 6 shows that the t-value and p-value for the pair of variables as follows: the physical symptoms (4.68, 0.00), emotions (7.37, 0.00), social status (6.86, 0.00), daily activities (2.66, 0.015), and self-esteem (3.40, 0.003). The results reflect a reduction of all means of the second data set, which reflects improvements in the nurse's conditions after 3–6 months after the COVID 19 infection except the daily activities, which increased in 3–6 months after the infection due to increasing of some activities, such as walking, exercises, and staying with family.

**Table 6:** The paired samples test

Pairs of variables		Paired Differences					t	df	Sig. (2-tailed)
		Mean	SD	Std. Error Mean	95% Confidence Interval of the Difference				
					Lower	Upper			
Pair 1	Physical symptoms (During—after)	.63	.63	.14	.35	.91	4.68	21	.000*
Pair 2	Emotion (During – after)	1.42	.90	.19	1.02	1.82	7.37	21	.000*
Pair 3	Social Status (During—after)	.84	.57	.12	.58	1.09	6.86	21	.000*
Pair 4	Daily activity (During – after)	.25	.44	.09	.055	.45	2.66	21	.015**
Pair 5	Self-esteem (During – after)	.72	.99	.21	.28	1.15	3.40	21	.003*

^*^P < 0.01, 0.05. **P > 0.05

Although the statistical difference among the two sets of data was significant, the authors calculated the effect size (i.e. Cohen's d [6]) to determine that the difference is meaningful. Table 7 shows that Cohen's d values of all pairs of variables were moderate (≥ 0.50) to large effects (≥ 0.80). Therefore, the results confirm that the sample size was sufficient.

**Table 7:** The effect size (Cohen's d)

	Variable	Mean (during)	Mean (after)		SD	Cohen's d
Pair 1	Physical symptoms (During—after)	1.85	1.22	0.63	0.63	1.00
Pair 2	Emotion (During – after)	2.77	1.35	1.42	0.90	1.58
Pair 3	Social Status (During—after)	1.90	1.07	0.83	0.57	1.46
Pair 4	Daily activity (During – after)	2.00	1.75	025	0.44	0.57
Pair 5	Self-esteem (During – after)	1.89	1.17	0.72	0.99	0.73

^*^d = 0.20 – 0.49 (small), d = 0.50 – 0.79 (medium), and d ≥ 0.80 (large) (Cohen, 1988)

### Analysis of the qualitative part

The authors conducted semi-structured interviews with a sample of 5 nurses who infected with the COVID 19 virus to explore their experience and main concerns during the infection time and after that, and explore the effect of the COVID 19 infection on their attitude, including emotions, beliefs, and behaviours toward a particular thing and future goals. The authors interviewed 5 nurses and stooped after reaching the state of data saturation.

The nurses like other people expressed tough experiences during the COVID 19 infection time. However, they mostly suffered psychologically. For example, one nurse expressed psychological stress when she saw her husband suffering to manage the house, shopping, taking care of 5 children, including food and schools. Another nurse stressed as she tried to avoid breastfeeding and care of her new-born baby; she said that “my baby also got the infection from me”. A third nurse said that "my fear was great since I was one of the first people contacted COVID 19 infection and no certain information was known about the disease at that time" and a fourth nurse said that "I was psychologically destroyed and terrified, it’s a challenge to suffer the extreme symptoms of the infection and at the same time worried about the kids in which you are helpless and can do nothing".

On the other hand, three nurses reported a perceived stigma; one nurse said "I felt the social stigma, and as a nurse, the stigma against me was stronger". Another nurse said "I felt the stigma, people were afraid to contact us to avoid the infection" and she also added that "I was worried about not be accepted between my colleagues at work and also between my friends in the community even after I tested negative". Furthermore, a nurse suffered from stigma at home; she said "I felt a stigma in my house and between my immediate family members, and my co-workers avoided me after recovery as I had bad cough".

Two nurses reported suffering from physical symptoms. Three nurses confirmed receiving support from managers, and Abu Dhabi Health Services Company (SEHA). Also, one nurse received support from some people who had been infected with the virus before and they shared their experiences and knowledge about the disease, how to manage symptoms with her.

Regarding lessons learned and future goals, one nurse pointed out that nurses should be careful and take COVID 19 seriously by adhering to all policies and procedures. Another said she benefited from the isolation to think about her life and review her objectives and plans. Another nurse also said she thought more about herself and the family. Two nurses did not address any impact of the COVID 19 infection period on their future goals. Lastly, one nurse pointed out that "inside me, I was enlightened that I have the power, the courage to beat COVID 19 infection. My beliefs, deep spiritual strengths, family support, and prayers helped me to cope with COVID 19 infection”. Another nurse blamed herself for being infected.

## Discussion

During the COVID 19 crisis, nurses were in two different situations; first health care providers and secondly, patients infected with COVID 19 virus. Accordingly, their experiences could be unique and rich. Therefore, this study aimed to determine the immediate and short-term physical, psychological, and social impact of COVID 19 infection on nurses and midwives retrospectively and find the effect of COVID 19 stigma on the self-esteem of the infected nurses.

In general, the majority of respondents rated most of the items in the physical, emotional, social, daily activities, and self-esteem scales very low, low, or moderate during the infection time. The symptoms improved significantly in 3–6 months after the infection. The results of the paired t-test confirmed a reduction of the means of the second data set of the physical, emotional, social, and self-esteem scales. Although the reduction in the mean of the second data set of the daily activities scale was insignificant, the authors still consider that a significant improvement in the symptoms as the second mean of the daily activities affected by contradicting effects of the increased level of some healthy activities, such as walking, exercises, and staying with family against unhealthy daily activities such as oversleeping, insomnia, and loss of appetite.

The results of the qualitative part of the study aimed to shed light on the result of the quantitative part. The nurses expressed that they suffered emotionally due to the separation from the family members, concerns about their safety, and inability to do their role as mothers toward their children. Also, the nurses felt stigma against them from people, colleagues, and some family members due to being infected with COVID 19 virus. However, the stigma did not affect significantly the items of the self-esteem scale. Thus, the stigma was not a concern among Tawam hospital infected nurses. Moreover, the nurses felt (psychologically destroyed, worried, and terrified) they felt helpless in regard the care for their own families. On the other hand, the nurses highlighted some lessons they learned during the infection time, such as being careful and adhering to all safety and protection policies and procedures, reviewing life objectives and plans, and focusing more on self and the family.

The study identified several factors that could be interacted to give positive outcomes in people infected with COVID 19 virus. Those factors included high self-efficacy, which was identified by Ramaci, et al., [23] as a factor that could lead to less fatigue and burnout, and more satisfaction amongst health care workers during COVID 19 crisis. The self-efficacy was reflected by using more positive coping mechanisms, such as praying, watching TV, staying with family, and practicing hobbies, and using less negative coping mechanisms, such as oversleeping, insomnia, smoking, or drinking alcohol. Furthermore, nursing knowledge and skills were an important factor in dealing with COVID 19 symptoms. On the other hand, psychological support from managers, families, and colleagues, as well as the high standards of care and treatment provided in UAE to patients infected with the COVID 19 virus, either in hospitals or at homes, were key factors for achieving such outcomes.

### Recommendation

We encourage governments to adopt the above-suggested model for all nurses infected with COVID 19 virus. This model could be useful, especially for those who have foreign nurses from deferent countries with no immediate family support. This could be achieved by introducing this model to the communities, providing enough teaching about the required nursing care, and assigning special telephone numbers to answer nurses concerns, and give them directions, support, and needed help. Support form nurses managers to their staff was very important in coping and adapting to the new situation.

Al last, the authors believe that nurse's experiences are unique and rich. Therefore, the authors encourage conducting more quantitative and qualitative researches to explore these experiences.

## Conclusion

The careful exploration of the experiences of nurses infected with the COVID 19 virus led to come up with a simple and clear model to manage nurses infected with COVID 19 in hospitals and at homes. The model highlights 4 key factors, including improving self-efficacy and nurses coping, providing the basic support to nurses and their families, providing continuous psychological support, and providing high standards of health care. This will maintain a positive status of wellbeing amongst infected nurses during the infection time and in 3–6 months after the infection. The stigma was not a concern for our nurses, on the other hand, at a certain time during the acute infection period nurses felt helpless to provide the support and help to their own families. Nurses felt strong, empowered, and positive by the great support nurses received from nursing administration at Tawam Hospital.

## Data Availability

The raw data generated and/or analysed during the current study ae not publicly available due to the institution policy to code and archive data in a central repository of the hospital, but data are available from the corresponding author on reasonable request by the editor.

## Abbreviations

ICN: International Council of Nurses
EPI-WIN: WHO’s Information Network for Epidemics
PTSD: Post-traumatic stress disorder
ISMA: International Stress Management Association
DOH: Department of Health
SEHA: Abu Dhabi Health Services Company
ICH: International Conference for Harmonization
GCP: Good Clinical Practice guidelines
UAE: United Arab Emirates
WHO: World Health Organization
